# All-cause mortality trends in patients hospitalized for atrial fibrillation in Sweden: Role of age, stroke risk, and education

**DOI:** 10.1016/j.ijcha.2022.101153

**Published:** 2022-11-26

**Authors:** Áron Sztaniszláv, Anders Magnuson, Ing-Liss Bryngelsson, Nils Edvardsson, Dritan Poci

**Affiliations:** a Department of Cardiology, Pulmonology and Clinical Physiology, School of Medical Sciences, Örebro University, Örebro, Sweden; b Clinical Epidemiology and Biostatistics, School of Medical Sciences, Örebro University, Örebro, Sweden; c Department of Occupational and Environmental Medicine, Faculty of Medicine and Health, Örebro University, Örebro, Sweden; d Sahlgrenska Academy at Sahlgrenska University Hospital, Gothenburg, Sweden; e Department of Clinical Physiology and Nuclear Medicine, Sahlgrenska University Hospital, Gothenburg, Sweden

**Keywords:** Atrial fibrillation, All-cause mortality, Stroke risk, Education level

## Abstract

**Background:**

The incidence of atrial fibrillation (AF) has long been increasing, and AF is associated with increased mortality. Over time, mortality trends may differ between subgroups depending on their underlying risk patterns and treatments.

**Aim:**

To explore all-cause-mortality trends over time in patients hospitalized for incident AF, and the effects of age, stroke risk, and education level.

**Methods and results:**

Patients hospitalized for incident AF between January 1995 and December 2003 were selected from Swedish national registries. Based on date of index admission, patients were divided into four cohorts and followed for five years. Age- and sex-matched controls were selected. Kaplan–Meier estimates and Cox regressions with trend analysis were used for statistical evaluation. There were 64,489 patients (mean age 72 ± 10.1 years) and 116,893 controls. There was a significantly decreasing trend in the relative risk of all-cause mortality in AF patients over time, with a trend hazard ratio of 0.94 (95 % confidence interval [CI] 0.92–0.96, *p* < 0.001) in women and 0.91 (95 % CI 0.89–0.93, *p* < 0.001) in men. The mortality trends did not differ significantly between AF patients and controls. The mortality risk remained unchanged in women aged 18–64 years, in patients with low stroke risk, and in patients with post-secondary education.

**Conclusion:**

The all-cause mortality risk decreased over time in both patients and controls, but subgroup analysis revealed an unchanged mortality trend in women aged 18–64 years, in patients with low stroke risk, and in patients with post-secondary education.

## 1 Introduction

Atrial fibrillation (AF) is the most common sustained arrythmia worldwide. AF incidence was estimated at 3406 per million people worldwide in 2017, representing a 17 % increase since 2007 and a 31 % increase since 1997. AF prevalence in Sweden was calculated to be approximately 2.9 % in the general population.[1], [2] A Danish cohort study found a 4 % annual increase in incidence over the 30-year study period (1983–2012), possibly explained by population ageing, improved survival of diseases leading to AF, higher awareness, enhanced diagnostic methods, and strict diagnostic criteria. [3].

AF is associated with mortality, being responsible for 0.51 % of global cumulative mortality, i.e., 0.287 million deaths worldwide in 2017, representing a 48 % increase since 2007. [4] In the Framingham Heart Study, the relative risk of mortality in AF patients was 1.5–1.9 over the 40-year follow-up period since 1948. [5].

In a previous report on AF-related all-cause mortality, we observed a 3–5-times higher mortality risk in an AF population, versus controls, over a 14-year follow-up period (adjusted hazard ratio [HR] 4.88, 95 % confidence interval [CI] 4.17–5.72 in women and adjusted HR 3.07, 95 % CI 2.82–3.35 in men); this risk was independent of age and investigated comorbidities. [6].

The aims of this study were to analyse the trends in five-year mortality risk over time in patients hospitalized with incident AF and to determine whether the mortality risk trend differed between subgroups stratified by age, stroke risk, and education level.

## 2 Methods

### 2.1 Registries and populations

This study was based on an earlier-described Swedish population and is an extended analysis of this earlier database. [6].

The patients were selected from the Swedish National Patient Registry (NPR), which is a nationwide database with full national coverage of all patients discharged from hospitals in Sweden. Sweden has a public health insurance system and healthcare is available to all people living in Sweden, so NPR data are highly valid. [7].

Patients treated in hospital between 1 January 1995 and 31 December 2008 and diagnosed with AF were identified in the NPR by the Epidemiological Centre at the Swedish National Board of Health and Welfare. Diagnostic data in these registries were coded using the International Classification of Diseases (ICD). The ICD-9 classification system was used between 1987 and 1997 and the ICD-10 system starting in 1997. AF was coded as 427 D (i.e., DA, DB, DC, DD, and DW) in ICD 9 (1987–1996) and as I 48.0 to I 48.9 (i.e., A, B, C, D, E, F, P, and X) in ICD 10 (starting in 1997). No distinction was made between paroxysmal, persistent, or permanent AF, and atrial flutter because of the risk of information bias.

Using the patient’s personal identification number, which contains information on date of birth and gender, Statistics Sweden (SCB) selected a control population from the linked database of NPR and General Population Registry (GPR) that did not have an AF diagnosis before or during the calendar years of inclusion. Two age- and gender-matched controls were selected for each AF patient.

The mortality endpoint and censoring information due to emigration were identified by linking records from the NPR, GPR, and Cause of Death Registry. The study database was anonymized by Swedish authorities before it was sent to the researchers. [6].

### 2.2 Study design

This is an observational retrospective cohort study investigating all-cause mortality as the outcome in the population of AF patients admitted to hospital over the nine years from January 1995 to December 2003. The included patients were hospitalized with AF as the primary diagnosis for the first time, with a confirmed absence of an AF diagnosis during the eight years preceding the index hospitalization. The patients were followed until death, emigration, or five years after the index hospitalization. The exclusion criteria were age under 18 years, cancer diagnosis (CC), chronic kidney disease (CKD), and chronic obstructive pulmonary disease (COPD) due to the high impact of these conditions on mortality. [8] The same selection criteria were used in the control group. The selection criteria were defined according to ICD-9 and ICD-10 codes. We excluded from the analysis patients who died within 30 days of the initial index hospitalization.

The nine-year inclusion period was divided into four 27-month periods, and the patients identified during each period formed one cohort according to the date of index hospitalization. The first cohort was used as the reference during the statistical analysis and comprised AF patients treated in hospital between 1 January 1995 and 31 March 1997. The hospitalization occurred between 1 April 1997 and 30 June 1999 in cohort 2, between 1 July 1999 and 30 September 2001 in cohort 3, and between 1 October 2001 and 31 December 2003 in cohort 4.

Any trends in mortality were compared with those of the matched controls.

### 2.3 Risk factors: Subpopulation analysis

Besides the AF diagnosis used as the inclusion criterion for patient selection, we collected data on all diseases registered during any hospitalization in both the AF population and the controls. We counted all comorbidities that occurred in the five years before and up to the time of the index event.

To investigate whether the same all-cause mortality risk trend could be observed in different subpopulations, we calculated the relative risk between each cohort compared with the reference cohort and the relative risk per time-period cohort in stratified groups of patients. Stratification was performed according to age, stroke risk, and education level. Three age categories were created, containing similar numbers of patients in order to maintain the statistical power of the analysis: 18–64, 65–74, and 75–86 years of age.

Stroke risk was evaluated by assessing the CHA_2_DS_2_-VASc score for patients and controls. [9] Stroke risk was also stratified in three categories: low-risk patients (CHA_2_DS_2_-VASc = 0), moderate-risk patients (CHA_2_DS_2_-VASc = 1), and high-risk patients (CHA_2_DS_2_-VASc ≥ 2). Statistical analysis was performed after stratifying for sex, so we ignored female sex as a risk factor for stroke.

The impact of education level on the mortality risk was studied as well, using GPR data. The education level categories were created based on the achieved education level: primary, secondary, and post-secondary education.

### 2.4 Statistical analysis

Unadjusted Kaplan–Meier plots were used to illustrate the cumulative all-cause mortality in the patient and control cohorts.

Cox regression models were used to compare the time to mortality between cohorts of AF patients, between matched cohorts of controls, and between the AF and matched control cohorts. The analysis was performed separately for sex when comparing the cohorts. In the Cox regression, time period was modelled as a categorical variable with the first period as the reference and using a linear variable (coded 0, 1, 2, 3) to estimate the trend in HR per time period. The regressions were adjusted for age in five-year categories from < 45 years, stroke risk defined as CHA_2_DS_2_-VASc (0–8), and education level (seven categories: primary education < 9 years, primary education ≥ 9 years, upper secondary education < 3 years, upper secondary education ≥ 3 years, post-secondary education < 3 years, post-secondary education ≥ 3 years, and post-graduate education). Due to missing education-level data, multiple imputation (MI) was implemented in STATA by MI command using multinomial regression and the above variables as independent variables. The imputation was repeated 25 times and the method was based on Rubin’s concept. [10].

Subgroup analyses with interaction tests were performed by stratifying on age, CHA_2_DS_2_-VASc score (0, 1 ≥ 2), and education level (primary education, secondary education, and post-secondary education). The non-proportional hazard assumption was evaluated using the phtest command in STATA with Schoenfeld residuals; if time periods showed evidence of non-proportionality, time-dependent analysis was conducted and the first year of follow-up was analysed and presented. The measure of association was the HR with 95 % CI. [11].

*P*-values below 0.05 were considered statistically significant. All statistical calculations were performed using STATA, release 14 (StataCorp, College Station, TX, USA). All data are available on request.

### 2.5 Ethics

This study complies with the Declaration of Helsinki, and the recommendations of the International Harmonization Conference Good Clinical Practice Guidelines. The study protocol was approved by the independent Regional Ethical Review Board in Uppsala, Sweden (Dnr 2009/273).

## 3 Results

### 3.1 Baseline population characteristics

In total, 64,489 AF patients (mean age 72 ± 10.1 years, 43.6 % women) met the inclusion criteria to participate in this study (Table 1). The cohorts had similar numbers of patients, ranging between 14,671 and 17,375. Regarding age, 81.9 % of the women and 58.5 % of the men were over 65 years old, and these proportions were similar in all cohorts. Regarding stroke risk, 65.5 % of the women and 45.9 % of the men had a stroke risk of CHA_2_DS_2_-VASc 2 or more.

**Table 1:** Characteristics of patients with AF, alive 30 days after inclusion and without cancer, COPD, and CKD at baseline.

	Total (n = 64489)	Cohort 1 (n = 14671)	Cohort 2 (n = 16574)	Cohort 3 (n = 17375)	Cohort 4 (n = 15869)
Sex, % women	43.6 %	45.0 %	43.9 %	42.8 %	42.8 %
Women (n)	**28,110**	**6600**	**7282**	**7430**	**6798**
Age, mean (SD)	72.5 (10.1)	72.3 (10.0)	72.5 (10.0)	72.7 (10.1)	72.6 (10.4)
Age categories, %					
<45	1.9 %	1.8 %	1.9 %	1.9 %	2.1 %
45–54	4.0 %	4.4 %	3.9 %	3.7 %	4.0 %
55–64	12.2 %	11.4 %	12.1 %	12.3 %	12.8 %
65–74	30.2 %	31.6 %	31.1 %	29.7 %	28.4 %
75–85	51.7 %	50.7 %	51.0 %	52.4 %	52.4 %
CHA_2_DS_2_–VASc score, %					
0	13.4 %	13.2 %	13.8 %	13.2 %	13.3 %
1	21.1 %	20.9 %	22.7 %	20.9 %	19.8 %
2–3	53.1 %	51.8 %	54.0 %	53.9 %	52.6 %
4 or more	12.4 %	14.1 %	9.4 %	12.0 %	13.3 %
Education (n)	**27,256**	**6299**	**7081**	**7235**	**6641**
Primary education < 9 years	56.3 %	61.3 %	58.5 %	55.1 %	50.6 %
Primary education ≥ 9 years	8.3 %	8.1 %	8.2 %	8.3 %	8.6 %
Upper secondary education < 3 years	22.1 %	19.6 %	20.9 %	23.0 %	24.7 %
Upper secondary education ≥ 3 years	3.0 %	2.4 %	3.0 %	3.1 %	3.4 %
Post–secondary education < 3 years	4.9 %	4.6 %	4.6 %	5.0 %	5.6 %
Post–secondary education ≥ 3 years	5.2 %	3.9 %	4.7 %	5.2 %	6.9 %
Post graduate	0.2 %	0.1 %	0.2 %	0.2 %	0.2 %
Men (n)	**36,379**	**8071**	**9292**	**9945**	**9071**
Age, mean (SD)	65.4 (13.0)	65.4 (12.9)	65.6 (13.0)	65.6 (12.9)	65.2 (13.0)
Age categories, %					
< 45	7.2 %	7.3 %	7.2 %	6.8 %	7.4 %
45–54	11.7 %	12.7 %	11.6 %	11.6 %	11.2 %
55–64	22.6 %	20.5 %	21.7 %	23.4 %	24.6 %
65–74	30.2 %	31.9 %	30.8 %	29.3 %	29.1 %
75–85	28.3 %	27.7 %	28.7 %	29.0 %	27.9 %
CHA_2_DS_2_–VASc score, %					
0	30.1 %	29.0 %	30.8 %	30.3 %	30.0 %
1	24.0 %	23.3 %	25.3 %	23.9 %	23.5 %
2–3	37.4 %	37.6 %	38.1 %	37.6 %	36.4 %
4 or more	8.5 %	10.1 %	5.8 %	8.1 %	10.1 %
Education (n)	**35,776**	**7853**	**9145**	**9823**	**8955**
Primary education < 9 years	41.2 %	47.1 %	42.6 %	40.0 %	35.9 %
Primary education ≥ 9 years	6.1 %	5.3 %	5.7 %	6.1 %	7.3 %
Upper secondary education < 3 years	21.4 %	20.5 %	21.1 %	21.8 %	22.0 %
Upper secondary education ≥ 3 years	13.5 %	12.2 %	13.2 %	13.6 %	15.0 %
Post–secondary education < 3 years	6.9 %	5.9 %	6.5 %	7.4 %	7.9 %
Post–secondary education ≥ 3 years	9.7 %	8.1 %	9.8 %	9.9 %	10.7 %
Post–graduate education	1.2 %	0.9 %	1.3 %	1.2 %	1.3 %

AF, atrial fibrillation; CKD, chronic kidney disease; COPD, chronic obstructive pulmonary disease; SD, standard deviation; we did not count with the point for the female gender in the CHA_2_DS_2_–VASc score because the data was stratified according to sex.

More women than men had primary education only (under nine years), i.e., 56.3 % and 41.2 %, respectively. Post-secondary or higher education was more common in men (17.8 % of AF patients and 17.5 % of controls) than women (10.3 % of AF patients and 11.1 % of controls).

The control group comprised 116,893 individuals, 42.7 % of whom were women (Supplemental Table 1). The mean age was 72.4 ± 10.3 years for the women and 64.8 ± 13.0 years for the men. The proportion of controls with moderate or high stroke risk was 53.9 % in the women and 31.3 % in the men, lower than the observed stroke risk in AF patients.

As in the patient group, in the control group men had higher education levels than did women, with 9.4 % versus 5.9 % having post-secondary and 1.2 % versus 0.2 % having post-graduate education, respectively.

### 3.2 Risk of all-cause mortality in AF patients and controls

Kaplan–Meier estimates and Cox regressions with trend analysis were used for statistical evaluation of the all-cause mortality. The unadjusted Kaplan–Meier analysis indicated higher cumulative mortality in AF patients than their controls during the five-year follow-up in every cohort. (Supplemental Figure 1 and 2) The all-cause mortality was higher in women than men in both the AF patients and the control population, displaying a decreasing trend according to time of the index hospitalization (53.2–44.0 per 1000 person-years in women vs 48.9–36.9 per 1000 person-years in men in AF patients, and 35.8–30.5 per 1000 person-years in women vs 34.1–25.6 per 1000 person-years in men in controls).

We found a statistically significant decreasing relative risk trend over time for all-cause mortality in AF patients (Table 2). The trend HR was 0.92 (95 % CI 0.90–0.94, *p* < 0.001) in both women and men per period and adjusted for age, CHA_2_DS_2_-VASc score, and education level. Except in cohort 2 in women, in which the HR was 1.03, the adjusted HR decreased relative to the reference cohort, being 0.93 (cohort 3) and 0.79 (cohort 4) in women and 0.98 (cohort 2), 0.88 (cohort 3), and 0.78 (cohort 4) in men. The MI-adjusted results were very similar. We found similar decreasing all-cause mortality risk trends over time in the controls: the adjusted HR was 0.93 (95 % CI 0.91–0.95, *p* < 0.001) per period for women and 0.92 (95 % CI 0.90–0.93, *p* < 0.001) for men. As a second step, we compared the mortality trends between AF patients and their controls. The trend analysis did not show any significant difference between AF patients and controls: the adjusted trend HR was 0.98 (95 % CI 0.95–1.02, *p* = 0.35) in women and 1.00 (95 % CI 0.97–1.03, *p* = 0.82) in men. (Supplemental Table 2).

**Table 2:** Cox regression for the outcome time to all–cause mortality comparing cohorts among AF patients and matched controls.

	n	Events	Rates	Unadjusted HR (95 % CI)	Adjusted 1 HR (95 % CI)	Adjusted 2 HR (95 % CI)	Adjusted 2 MI HR (95 % CI)
Women AF (n)				**28,110**	**28,110**	**27,256**	**28,110**
Cohort 1	6600	1545	53.2	reference cohort	reference cohort	reference cohort	reference cohort
Cohort 2	7282	1662	51.4	0.96 (0.90–1.04)	1.02 (0.95–1.09)	1.03 (0.96–1.10)	1.03 (0.96–1.10)
Cohort 3	7430	1642	49.6	0.93 (0.87–0.99)	0.90 (0.84–0.97)	0.93 (0.86–0.99)	0.92 (0.86–0.99)
Cohort 4	6798	1352	44.0	0.82 (0.77–0.89)^1^	0.76 (0.71–0.82)	0.79 (0.73–0.85)	0.78 (0.73–0.84)
Trend, HR per period				0.94 (0.92–0.96)^1^ *p* < 0.001	0.91 (0.89–0.93) *p* < 0.001	0.92 (0.90–0.94) *p* < 0.001	0.92 (0.90–0.94) *p* < 0.001
Women controls (*n*)				**49,948**	**49,948**	**48,151**	**49,948**
Cohort 1	12,070	1,989	35.8	reference cohort	reference cohort	reference cohort	reference cohort
Cohort 2	13,068	2,030	33.5	0.94 (0.88–0.99)	1.01 (0.95–1.07)	1.02 (0.95–1.09)	1.01 (0.95–1.08)
Cohort 3	13,048	1,945	32.1	0.90 (0.84–0.95)	0.88 (0.82–0.93)	0.91 (0.85–0.97)	0.89 (0.83–0.94)
Cohort 4	11,762	1,675	30.5	0.85 (0.80–0.91)	0.79 (0.74–0.84)	0.82 (0.76–0.87)	0.80 (0.75–0.86)
Trend, HR per period				0.95 (0.93–0.97) *p* < 0.001	0.92 (0.90–0.94) *p* < 0.001	0.93 (0.91–0.95) *p* < 0.001	0.92 (0.91–0.94) *p* < 0.001
Men AF (*n)*				**36,379**	**36,379**	**35,776**	**36,379**
Cohort 1	8071	1746	48.9	reference cohort	reference cohort	reference cohort	reference cohort
Cohort 2	9292	1819	43.5	0.89 (0.83–0.95)	0.94 (0.88–1.00)	0.98 (0.92–1.05)	0.95 (0.89–1.02)
Cohort 3	9945	1848	41.1	0.84 (0.79–0.90)	0.83 (0.77–0.88)	0.88 (0.82–0.94)	0.85 (0.79–0.91)
Cohort 4	9071	1537	36.9	0.76 (0.70–0.81)^1^	0.73 (0.68–0.78)^1^	0.78 (0.73–0.84)^2^	0.76 (0.70–0.81)
Trend, HR per period				0.91 (0.89–0.93)^1^ *p* < 0.001	0.90 (0.88–0.92)^1^ *p* < 0.001	0.92 (0.90–0.94)^2^ *p* < 0.001	0.91 (0.89–0.93) *p* < 0.001
Men controls (*n*)				**66,945**	**66,945**	**65,503**	**66,945**
Cohort 1	14,982	2351	34.1	reference cohort	reference cohort	reference cohort	reference cohort
Cohort 2	17,198	2503	31.3	0.92 (0.87–0.97)	0.99 (0.94–1.05)	1.00 (0.95–1.06)	1.00 (0.94–1.06)
Cohort 3	18,260	2527	29.7	0.87 (0.82–0.92)	0.86 (0.81–0.91)	0.89 (0.84–0.95)	0.88 (0.83–0.93)
Cohort 4	16,505	1989	25.6	0.75 (0.71–0.80)	0.74 (0.69–0.78)	0.77 (0.73–0.82)	0.76 (0.71–0.80)
Trend, HR per period				0.91 (0.90–0.93) *p* < 0.001	0.90 (0.88–0.92) *p* < 0.001	0.92 (0.90–0.93) *p* < 0.001	0.91 (0.89–0.93) *p* < 0.001

AF, atrial fibrillation; CI, confidence interval; HR, hazard ratio; MI, multiple imputation; rates per 1000 person–years; Adjusted 1, adjusted for age (in five–year categories from < 45 years) and CHA_2_DS_2_–VASc score (0–8); Adjusted 2, adjusted for age (in five–year categories from < 45 years), CHA_2_DS_2_–VASc score (0–8), and education level; MI, multiple imputation.

1 Non–proportional hazard; not tested in MI model.

2 Non–proportional hazard, in the first year of follow–up; HR = 0.87 (95 % CI 0.76–1.01) for Cohort 2, HR = 0.81 (95 % CI 0.70–0.93) for Cohort 3, HR = 0.59 (95 % CI 0.50–0.69) for Cohort 4 relative to reference period; trend HR = 0.85 (95 % CI 0.81–0.89, *p* < 0.001) per period.

### 3.3 Heterogeneity of mortality risk

#### 3.3.1 Stratification according to age.

Statistically significant decreasing mortality risk trends, similar to those in the non-stratified analysis, were noted within age groups in men (aged 18–64, 65–74, and 75–85 years) and in older women (aged 65–74 and 75–85 years) (Supplemental Table 3). In women aged 18–64 years, the adjusted HR was 0.96 (95 % CI 0.85–1.08) per period. However, this patient group was relatively small with few mortality cases, so the result should be interpreted with caution.

**Table 3:** Trend HR values of stratified Cox regression analysis for outcome time to all-cause mortality comparing cohorts of AF patients.

Age stratified adjusted regression Trend HR (95 % CI)			Stroke risk stratified adjusted regression Trend HR (95 % CI)			Education level stratified adjusted regression Trend HR (95 % CI)		
Age	HR men	HR women	Stroke risk	HR men	HR women	Education level	HR men	HR Women
18–64 years	0.93 (0.88–0.99) p = 0.027	0.96 (0.85–1.08) p = 0.49	Low	0.95 (0.87–1.05) p = 0.3	1.08 (0.92–1.26) p = 0.36	Primary School	0.91 (0.88–0.93) p < 0.001^3^	0.92 (0.90–0.95) p < 0.001
65–74 years	0.92 (0.88–0.96) p < 0.001	0.90 (0.85–0.95) p < 0.001	Medium	0.93 (0.88–0.98) p < 0.010	0.86 (0.80–0.94) p < 0.001	Secondary School	0.91 (0.87–0.94) p < 0.001	0.91 (0.86–0.97) p = 0.001
75–85 years	0.91 (0.89–0.94) p < 0.001^1^	0.93 (0.90–0.95) p < 0.001	High	0.91 (0.89–0.94) p < 0.001^2^	0.92 (0.90–0.95) p < 0.001	Post-Secondary School	1.04 (0.96–1.12) p = 0.32	0.93 (0.83–1.04) p = 0.23

AF, atrial fibrillation; CI, confidence interval; HR, hazard ratio; rates per 1000 person–years; Age stratified Cox-regression was adjusted for for age (in five–year categories from < 45 years), CHA_2_DS_2_–VASc score (0–8), and education level.

Stroke risk stratified Cox-regression was adjusted for age (in five–year categories from < 45 years), CHADS_2_–VA_2_Sc score (0–8), and education level.

Education level stratified Cox-regression was adjusted for age (in five–year categories from < 45 years) and CHA_2_DS_2_–VASc score (0–8).

1 Non–proportional hazard.

2 Non–proportional hazard, in the first year of follow–up; HR = 0.88 (95 % CI 0.79–1.04) for Cohort 2, HR = 0.81 (95 % CI 0.69–0.95) for Cohort 3, HR = 0.58 (95 % CI 0.48–0.69) for Cohort 4 relative to reference period; trend HR 0.85 (95 % CI 0.80–0.89, *p* < 0.001) per period.

3 Non–proportional hazard, in the first year of follow–up; HR = 0.97 (95 % CI 0.81–1.15) for Cohort 2, HR = 0.78 (95 % CI 0.65–0.93) for Cohort 3, HR = 0.56 (95 % CI 0.46–0.69) for Cohort 4 relative to reference period; trend HR 0.83 (95 % CI 0.78–0.88, *p* < 0.001) per period.

#### 3.3.2 Stratification according to stroke risk.

Statistically significant decreasing trends in all-cause mortality risk were seen in patients with moderate and high CHA_2_DS_2_-VASc in both men and women, but no significant mortality trend was noted in the low-stroke-risk population in either men or women (Supplemental Table 4). In female patients with low stroke risk, a small non-significant increasing trend in mortality risk was noted: the adjusted HR was 1.08 (95 % CI 0.92–1.26, *p* = 0.36) per period, which was significantly different from the risk trend for women with moderate stroke risk (interaction test *p* = 0.025), for whom the adjusted HR was 0.86 (95 % CI 0.80–0.94, *p* < 0.001). In men with AF and low stroke risk, the adjusted mortality risk trend HR was 0.95 (95 % CI 0.87–1.05, *p* = 0.30) per period, not significantly different from that of men with moderate or high stroke risk, as indicated by the interaction test.

#### 3.3.3 Stratification according to education level.

Significantly decreasing adjusted mortality risk trends were observed in male and female AF patients with primary and secondary education (Supplemental Table 5). However, in female AF patients with post-secondary education, the adjusted trend HR was unchanged at 0.93 (95 % CI 0.83–1.04, *p* = 0.23). In male AF patients with post-secondary education, the adjusted mortality risk trend HR was 1.04 (95 % CI 0.96–1.12), significantly different from the decreasing mortality trends in male AF patients with primary (interaction test *p* = 0.001) and secondary (interaction test *p* = 0.002) education.

## 4 Discussion

The all-cause mortality risk was higher in AF patients than in the matched controls. The AF patients displayed a higher all-cause mortality rate during the first year of follow-up, but without effects on the mortality trend over time. As expected, the proportion of patients with moderate or high stroke risk was higher in AF patients.

There was a statistically significant decreasing trend in the risk of all-cause mortality in the AF patients according to the time of the first AF-related hospitalization. We observed a similar decreasing trend in the control population, although it did not differ significantly from the observed trends in AF patients. The mortality risk remained unchanged, however, in women patients aged 18–69 years, in patients with low stroke risk, and in patients with post-secondary education during the follow-up.

### 4.1 Decreasing all-cause mortality over time

All-cause mortality particularly increased in AF patients during the first three to four months after the initial diagnosis. (5)(6)(8) In a report from Olmstead County covering first-ever AF or atrial flutter between 2000 and 2010, the AF incidence and survival remained constant over the studied decade, while a dramatic excess mortality risk was observed within 90 days of the AF diagnosis, emphasizing the importance of early risk stratification. [12] The same pattern of mortality risk was described in a recent report on the GARFIELD-AF registry. Contrary to the findings from GARFIELD-AF, our results showed that the mortality risk of patients with low stroke risk was higher than the mortality risk of the respective matched controls, which is in line with data from the Framingham Heart Study. [13].

Reports from the USA and Denmark have shown a decreasing all-cause mortality trend despite increasing incidence, resulting in a higher prevalence of AF. [14], [15] Despite the increased incidence and prevalence of AF, the stroke risk decreased by 74 % and the all-cause mortality risk decreased by 25.4 % during the examined 50 years. A 30-year nationwide Danish observational study followed patients with first-time hospital-diagnosed AF, finding that the one- and five-year mortality had declined by more than 40 % over the preceding three decades in patients with all levels of comorbidity. (3).

Factors that might explain the decreasing mortality trend in AF patients over time include the validation and implementation of different clinical risk scores for stroke prediction, [16] new information on rhythm- and rate-control treatment strategies and on the role of risk factors, [17], [18] and the dissemination and application of international guidelines for anticoagulation therapy and/or the use of pharmacological or invasive treatment of AF. Adherence to clinical recommendations regarding anticoagulation therapy was described as less than adequate during the study period, but improvements over time are also reported as evidence of how the management of patients differed. [19], [20].

A recent study analysing the mortality-to-incidence ratios (MIRs) in AF patients across Europe found no improvement from 1990 to 2017 despite advances in AF management. That study describes the AF-related mortality, and the authors supposed that the increase in MIRs was caused by the survivor effect, i.e., the improved survival of different comorbidities probably leads to mortality connected to AF. [21].

The AFFIRM study found no significant mortality difference between the rhythm- and rate-control treatment strategies in AF. [17] The cause-specific mortality analysis in that study showed that mortality from vascular causes, such as stroke, occurred in only 3 % of patients in both treatment groups, and that oral anticoagulation therapy was often discontinued in the rhythm-control arm. The authors suggested that the already low mortality rate for vascular events could not be further improved with the use of rhythm-control medication. [22] The recent randomized EAST-AFNET 4 study examined the effect of early rhythm-control treatment versus that of the more conservative rate-control medication. The cardiovascular mortality was examined as part of a composite primary endpoint. During the 5.1-year follow-up period, the cardiovascular mortality was significantly lower in the rhythm-control group. However the all-cause mortality, which was examined as a primary safety endpoint, did not differ significantly despite anticoagulation therapy, even though the majority of the patients had it (91.2 % in the early rhythm-control group and 89.7 % in the usual care group). [23].

The use of oral anticoagulation therapy was supported by well-known evidence of improved survival. [24] meta-analyses and registry studies have confirmed that the main causes of mortality in anticoagulated AF patients are heart failure (HF), coronary artery disease (CAD), and myocardial infarction (MI). [25].

Ischaemic and haemorrhagic strokes were the cause of death in 5.6 % and 5.7 % of AF patients, respectively.

A recent analysis of the AFNET database, similarly to other observational studies, showed that non-cardiovascular diseases such as chronic kidney disease, diabetes mellitus, chronic obstructive pulmonary disease, and peripheral artery disease play a key role in the mortality of AF patients even now, during the direct oral anticoagulant (DOAC) era. [26], [27], [28].

Our results showing a decreasing all-cause mortality risk trend in both the AF and control groups support the hypothesis that this change might be the result of a combination of better treatment options for HF, CAD, and other cardiovascular or non-cardiovascular comorbidities. Improved anticoagulation and rhythm control do not seem to be decisive factors influencing the mortality risk in AF patients, emphasizing the importance of integrated AF management.

### 4.2 Unchanged all-cause mortality risk trends in subgroups

The consistent decreasing trend in mortality risk was not observed during the subgroup analysis of younger patients aged 18–64 years, those with low stroke risk, and those with higher education, in whom the trends were unchanged during follow-up. (Table 3) These patients are usually considered to have lower stroke and mortality risks. [29] In our study, the mortality rates were relatively low in these subpopulations, which presumably could have led to the non-significant change in mortality risk trends. In the case of men with low stroke risk, the decreasing mortality risk trend disappeared after adjusting for education level, and the mortality risk remained largely unchanged or slightly increased in patients with higher education. However, the low event rates probably indicate that the prognosis of these patients could not be improved despite the medical armamentarium during the study period.

## 5 Strengths and limitations

The strengths of this study are that its analyses and results are based on a large and complete consecutive nationwide population of patients hospitalized with incident AF. Each included patient had almost two matched controls. We did not try to distinguish between AF and atrial flutter or between AF types. The included patients had a first-ever hospital diagnosis of AF, which does not exclude that AF may have been diagnosed in an outpatient clinic or a primary healthcare facility. However, the exclusion of patients diagnosed during the eight years before the study period increases the probability of AF being truly incident in our study population.

In the examined period there wasńt any consecutive national database on the prescribed medications in Sweden therefore it wasńt possible to analyse the effect of the anticoagulation or other drug therapy on the all-cause mortality risk. The Swedish National Prescribed Drug Registry was established in 2005.

## 6 Conclusion

The all-cause mortality risk of patients hospitalized for incident AF was higher than in their controls. The HRs for all-cause mortality declined over time, although this trend did not differ significantly between the AF patients and their controls. We found an unchanged mortality trend in women aged 18–64 years, in patients with lower stroke risk, and in patients with post-secondary education.

## Declaration of Competing Interest

The authors declare that they have no known competing financial interests or personal relationships that could have appeared to influence the work reported in this paper.
