# Supplementary figures and images for: All-cause mortality trends in patients hospitalized for atrial fibrillation in Sweden: Role of age, stroke risk, and education
Source: Int J Cardiol Heart Vasc. 2022 Nov 26;43:101153. doi: 10.1016/j.ijcha.2022.101153 (PMC9706152; doi:10.1016/j.ijcha.2022.101153)

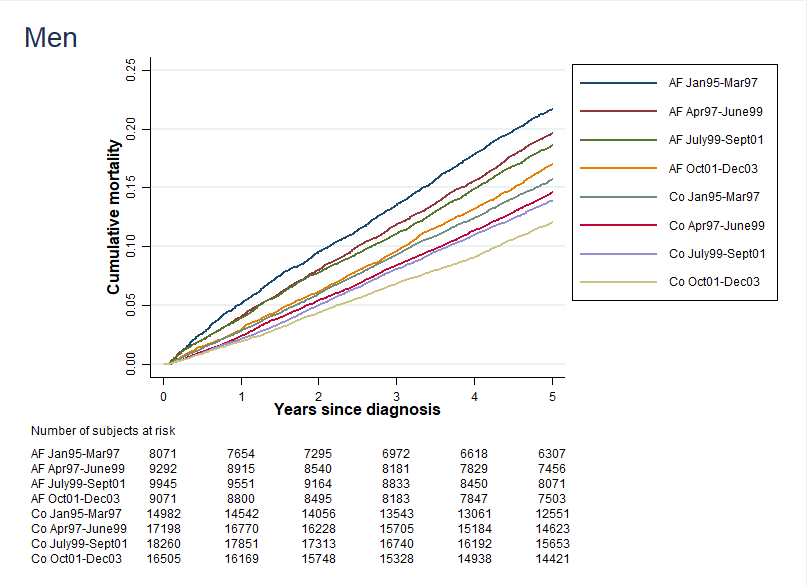


Supplemental Figure 1 Cumulative mortality according to Kaplan – Meier analysis in men

Supplement: Supplementary data 1 [file mmc1.docx]

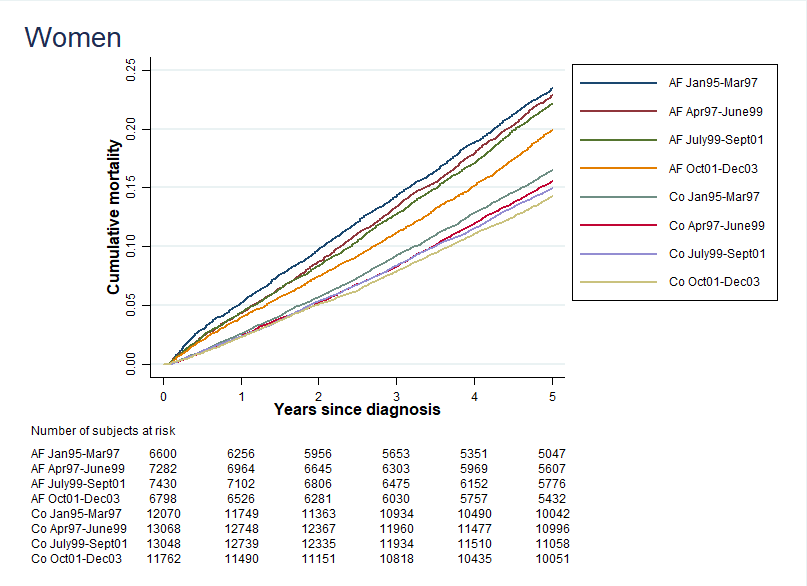


Supplemental Figure 2 Cumulative mortality according to Kaplan – Meier analysis in women

Supplement: Supplementary data 2 [file mmc2.docx]
