# Supplementary material for: All-cause mortality trends in patients hospitalized for atrial fibrillation in Sweden: Role of age, stroke risk, and education
Source: Int J Cardiol Heart Vasc. 2022 Nov 26;43:101153. doi: 10.1016/j.ijcha.2022.101153 (PMC9706152; doi:10.1016/j.ijcha.2022.101153)
Supplement: Supplementary data 3 [file mmc3.docx]

**Supplemental Table 1** Characteristics of controls, alive 30 days after inclusion and without cancer, COPD, and CKD at baseline.

|  | Total  (n=116893) | Jan 1995 – March 1997  (n=27052) | Apr 1997 – June 1999  (n=30266) | July 1999 – Sept 2001  (n=31308) | Oct 2001 – Dec 2003  (n=28267) |
| --- | --- | --- | --- | --- | --- |
|  |  |  |  |  |  |
| Sex, % women | 42.7% | 44.6% | 43.2% | 41.7% | 41.6% |
| Women (n) | **49948** | **12070** | **13068** | **13048** | **11762** |
| Age, mean (SD) | 72.4 (10.3) | 72.3 (10.1) | 72.3 (10.1) | 72.5 (10.3) | 72.4 (10.6) |
| Age categories, % |  |  |  |  |  |
| <45 | 2.1% | 2.0% | 2.1% | 2.1% | 2.3% |
| 45–54 | 4.0% | 4.4% | 3.9% | 3.8% | 4.1% |
| 55–64 | 12.3% | 11.7% | 12.3% | 12.4% | 12.8% |
| 65–74 | 30.4% | 31.7% | 31.4% | 29.9% | 28.5% |
| 75–85 | 51.2% | 50.3% | 50.4% | 51.8% | 52.2% |
| CHA_2_DS_2_–VASc score, % |  |  |  |  |  |
| 0 | 17.8% | 17.3% | 17.9% | 17.7% | 18.5% |
| 1 | 28.2% | 28.7% | 29.8% | 28.0% | 26.2% |
| 2–3 | 50.0% | 49.4% | 50.5% | 50.3% | 50.0% |
| 4 or more | 3.9% | 4.6% | 1.8% | 4.1% | 5.3% |
| Education (n) | **48151** | **11411** | **12640** | **12663** | **11437** |
| Primary education <9 years | 54.3% | 59.0% | 56.2% | 53.0% | 49.2% |
| Primary education ≥9 years | 8.5% | 8.5% | 8.4% | 8.4% | 8.5% |
| Upper secondary education <3 years | 22.8% | 21.0% | 22.1% | 23.5% | 24.4% |
| Upper secondary education ≥3 years | 3.5% | 2.8% | 3.1% | 3.7% | 4.4% |
| Post–secondary education <3 years | 5.0% | 3.9% | 4.7% | 5.4% | 5.9% |
| Post–secondary education ≥3 years | 5.9% | 4.7% | 5.4% | 5.9% | 7.4% |
| Post graduate | 0.2% | 0.1% | 0.1% | 0.2% | 0.2% |
| Men (n) | **66945** | **14982** | **17198** | **18260** | **16505** |
| Age, mean (SD) | 64.8 (13.0) | 64.8 (13.0) | 64.9 (13.0) | 64.9 (13.0) | 64.5 (13.1) |
| Age categories, % |  |  |  |  |  |
| <45 | 7.7% | 7.8% | 7.7% | 7.3% | 8.0% |
| 45–54 | 12.5% | 13.4% | 12.3% | 12.3% | 11.9% |
| 55–64 | 23.6% | 21.4% | 22.7% | 24.5% | 25.7% |
| 65–74 | 30.0% | 31.7% | 30.8% | 29.1% | 28.8% |
| 75–85 | 26.2% | 25.8% | 26.5% | 26.8% | 25.5% |
| CHA_2_DS_2_–VASc score, % |  |  |  |  |  |
| 0 | 41.5% | 39.8% | 41.3% | 41.7% | 42.8% |
| 1 | 27.2% | 27.9% | 28.9% | 26.4% | 25.8% |
| 2–3 | 28.1% | 28.3% | 28.2% | 28.7% | 27.2% |
| 4 or more | 3.2% | 3.4% | 1.6% | 3.2% | 4.1% |
| Education (n) | **65503** | **14527** | **16821** | **17918** | **16237** |
| Primary education <9 years | 40.9% | 46.1% | 43.2% | 39.3% | 35.8% |
| Primary education ≥9 years | 6.5% | 5.7% | 6.0% | 6.8% | 7.3% |
| Upper secondary education <3 years | 21.8% | 20.1% | 21.2% | 22.1% | 22.9% |
| Upper secondary education ≥3 years | 13.4% | 12.9% | 12.8% | 13.8% | 14.2% |
| Post–secondary education <3 years | 6.9% | 5.9% | 6.1% | 7.4% | 8.1% |
| Post–secondary education ≥3 years | 9.4% | 7.8% | 9.5% | 9.6% | 10.4% |
| Post graduate | 1.2% | 1.0% | 1.1% | 1.2% | 1.3% |

CKD, chronic kidney disease; COPD, chronic obstructive pulmonary disease; SD, standard deviation; we did not count with the point for the female gender in the CHA_2_DS_2_–VASc score because the data was stratified according to sex.
