# Supplementary material for: All-cause mortality trends in patients hospitalized for atrial fibrillation in Sweden: Role of age, stroke risk, and education
Source: Int J Cardiol Heart Vasc. 2022 Nov 26;43:101153. doi: 10.1016/j.ijcha.2022.101153 (PMC9706152; doi:10.1016/j.ijcha.2022.101153)
Supplement: Supplementary data 4 [file mmc4.docx]

**Supplemental Table 2** Cox regression for the outcome time to all–cause mortality comparing AF patients and controls (only AF patients and controls alive 30 days after inclusion and without cancer, COPD, and CKD).

|  | AF vs. Controls | | |
| --- | --- | --- | --- |
|  | Unadjusted  HR (95% CI) | Adjusted 1  HR (95% CI) | Adjusted 2  HR (95% CI) |
| Women |  |  |  |
| Jan 1995 ­– Mar 1997 | 1.49 (1.40–1.60)^1^ | 1.18 (1.10–1.26)^1^ | 1.19 (1.11–1.28) |
| Apr 1997 – June 1999 | 1.54 (1.44–1.64)^1^ | 1.22 (1.14–1.31) | 1.23 (1.15–1.32) |
| July 1999 – Sept 2001 | 1.55 (1.45–1.65) | 1.22 (1.14–1.30) | 1.22 (1.14–1.31) |
| Oct 2001 – Dec 2003 | 1.44 (1.34–1.55) | 1.13 (1.05–1.22) | 1.14 (1.05–1.23) |
| Trend, HR per period | 0.99 (0.96–1.02)  *p*=0.59 | 0.99 (0.96–1.02)  *p*=0.47 | 0.98 (0.95–1.02)  *p*=0.35 |
| Men |  |  |  |
| Jan 1995 – Mar 1997 | 1.44 (1.35–1.53)^1^ | 1.12 (1.06–1.20) | 1.12 (1.05–1.20) |
| Apr 1997 – June 1999 | 1.39 (1.31–1.48)^1^ | 1.09 (1.02–1.16) | 1.12 (1.05–1.19) |
| July 1999 – Sept 2001 | 1.38 (1.30–1.47)^1^ | 1.08 (1.02–1.15) | 1.10 (1.03–1.17) |
| Oct 2001 – Dec 2003 | 1.44 (1.35–1.54) | 1.11 (1.03–1.18) | 1.12 (1.05–1.20) |
| Trend, HR per period | 1.00 (0.97–1.03)  *p*=0.98 | 0.99 (0.97–1.02)  *p*=0.67 | 1.00 (0.97–1.03)  *p*=0.82 |
|  |  |  |  |

AF, atrial fibrillation; CI, confidence interval; CKD, chronic kidney disease; COPD, chronic obstructive pulmonary disease; HR, hazard ratio; Adjusted 1, adjusted for age (in five–year categories from <45 years) and CHA_2_DS_2_–VASc score (0–8); Adjusted 2, adjusted for age (in five–year categories from <45 years), CHA_2_DS_2_–VASc score (0–8), and education level.

^1^ Non–proportional hazard.
