# Supplementary material for: All-cause mortality trends in patients hospitalized for atrial fibrillation in Sweden: Role of age, stroke risk, and education
Source: Int J Cardiol Heart Vasc. 2022 Nov 26;43:101153. doi: 10.1016/j.ijcha.2022.101153 (PMC9706152; doi:10.1016/j.ijcha.2022.101153)
Supplement: Supplementary data 5 [file mmc5.docx]

**Supplemental Table 3** Age–stratified cohorts: Cox regression for the outcome time to all–cause mortality comparing AF patients with matched controls; rates per 1000 person–years.

|  | n | Events | Rates | Unadjusted  HR (95% CI) | Adjusted 1  HR (95% CI) | Adjusted 2  HR (95% CI) |
| --- | --- | --- | --- | --- | --- | --- |
| Women AF |  |  |  |  |  |  |
| Age 18 – 64 years |  |  |  |  |  |  |
| Cohort 1 | 1168 | 64 | 11.3 | reference cohort | reference cohort | reference cohort |
| Cohort 2 | 1300 | 71 | 11.2 | 1.00 (0.71–1.40) | 1.03 (0.73–1.44) | 1.02 (0.71–1.47) |
| Cohort 3 | 1331 | 78 | 12.1 | 1.08 (0.77–1.50) | 1.06 (0.76–1.48) | 1.27 (0.90–1.80) |
| Cohort 4 | 1286 | 49 | 7.8 | 0.69 (0.47–1.00) | 0.64 (0.44–0.94) | 0.78 (0.53–1.16) |
| Trend, HR per period |  |  |  | 0.91 (0.82–1.02)  *p*=0.099 | 0.89 (0.80–0.99)  *p*=0.040 | 0.96 (0.85–1.08)  *p*=0.49 |
| Age 65 – 74 years |  |  |  |  |  |  |
| Cohort 1 | 2088 | 324 | 33.8 | reference cohort | reference cohort | reference cohort |
| Cohort 2 | 2267 | 295 | 27.8 | 0.82 (0.70–0.96) | 0.89 (0.76–1.04) | 0.89 (0.76–1.05) |
| Cohort 3 | 2205 | 281 | 27.2 | 0.80 (0.68–0.94) | 0.82 (0.70–0.96) | 0.85 (0.72–1.01) |
| Cohort 4 | 1933 | 213 | 23.3 | 0.69 (0.58–0.82) | 0.68 (0.57–0.80) | 0.72 (0.60–0.85) |
| Trend, HR per period |  |  |  | 0.89 (0.84–0.94)  *p*<0.001 | 0.88 (0.84–0.93)  *p*<0.001 | 0.90 (0.85–0.95)  *p*<0.001 |
| Age 75 – 85 years |  |  |  |  |  |  |
| Cohort 1 | 3344 | 1157 | 84.1 | reference cohort | reference cohort | reference cohort |
| Cohort 2 | 3715 | 1296 | 84.2 | 1.00 (0.92–1.08) | 1.06 (0.97–1.14) | 1.07 (0.98–1.16) |
| Cohort 3 | 3894 | 1283 | 78.4 | 0.93 (0.86–1.01) | 0.92 (0.85–0.99) | 0.94 (0.86–1.02) |
| Cohort 4 | 3579 | 1090 | 71.3 | 0.84 (0.78–0.92) | 0.79 (0.73–0.86) | 0.81 (0.74–0.88) |
| Trend, HR per period |  |  |  | 0.94 (0.92–0.97)  *p*<0.001 | 0.92 (0.90–0.94)  *p*<0.001 | 0.93 (0.90–0.95)  *p*<0.001 |
| Interaction tests for trend |  |  |  |  |  |  |
| Age 18 – 64 vs. 65 – 74 |  |  |  | *p*=0.73 | *p*=0.94 | *p*=0.56 |
| Age 18 – 64 vs. 75 – 85 |  |  |  | *p*=0.54 | *p*=0.58 | *p*=0.83 |
| Age 65 – 74 vs. 75 – 85 |  |  |  | *p*=0.062 | *p*=0.23 | *p*=0.42 |
| Men AF |  |  |  |  |  |  |
| Age 18 – 64 years |  |  |  |  |  |  |
| Cohort 1 | 3262 | 237 | 15.1 | reference cohort | reference cohort | reference cohort |
| Cohort 2 | 3764 | 221 | 12.1 | 0.80 (0.67–0.96) | 0.86 (0.72–1.04) | 1.00 (0.82–1.21) |
| Cohort 3 | 4149 | 271 | 13.5 | 0.89 (0.75–1.06) | 0.91 (0.76–1.08) | 1.08 (0.90–1.31) |
| Cohort 4 | 3906 | 187 | 9.8 | 0.65 (0.53–0.79) | 0.62 (0.51–0.75) | 0.76 (0.62–0.93) |
| Trend, HR per period |  |  |  | 0.89 (0.84–0.95)  *p*<0.001 | 0.87 (0.82–0.93)  *p*<0.001 | 0.93 (0.88–0.99)  *p*=0.027 |
| Age 65 – 74 years |  |  |  |  |  |  |
| Cohort 1 | 2576 | 522 | 45.3 | reference cohort | reference cohort | reference cohort |
| Cohort 2 | 2865 | 542 | 41.8 | 0.92 (0.82–1.04) | 1.01 (0.89–1.14) | 1.04 (0.92–1.18) |
| Cohort 3 | 2910 | 489 | 36.7 | 0.81 (0.71–0.92) | 0.83 (0.74–0.94) | 0.88 (0.77–0.99) |
| Cohort 4 | 2637 | 410 | 33.7 | 0.74 (0.65–0.84) | 0.74 (0.65–0.84) | 0.80 (0.70–0.91) |
| Trend, HR per period |  |  |  | 0.90 (0.87–0.94)  *p*<0.001 | 0.90 (0.86–0.93)  *p*<0.001 | 0.92 (0.88–0.96)  *p*<0.001 |
| Age 75 – 85 years |  |  |  |  |  |  |
| Cohort 1 | 2233 | 987 | 115.6 | reference cohort | reference cohort | reference cohort |
| Cohort 2 | 2663 | 1,056 | 99.2 | 0.86 (0.78–0.93) | 0.92 (0.84–1.00) | 0.95 (0.87–1.04) |
| Cohort 3 | 2886 | 1,088 | 93.5 | 0.81 (0.74–0.88) | 0.80 (0.74–0.88) | 0.83 (0.76–0.91) |
| Cohort 4 | 2528 | 940 | 90.4 | 0.78 (0.71–0.85)* | 0.75 (0.69–0.82)* | 0.78 (0.71–0.85)* |
| Trend, HR per period |  |  |  | 0.92 (0.90–0.95)*  *p*<0.001 | 0.90 (0.88–0.93)*  *p*<0.001 | 0.91 (0.89–0.94)*  p<0.001 |
| Interaction tests for trend |  |  |  |  |  |  |
| Age 18 – 64 vs. 65 – 74 |  |  |  | *p*=0.75 | *p*=0.47 | *p*=0.81 |
| Age 18 – 64 vs. 75 –85 |  |  |  | *p*=0.32 | *p*=0.28 | *p*=0.83 |
| Age 65 – 74 vs. 75 – 85 |  |  |  | *p*=0.38 | *p*=0.71 | *p*=0.95 |

AF, atrial fibrillation; CI, confidence interval; HR, hazard ratios; Adjusted 1, adjusted for age (in five–year categories from <45 years) and CHA_2_DS_2_–VASc score (0–8); Adjusted 2, adjusted for age (in five–year categories from <45 years), CHA_2_DS_2_–VASc score (0–8), and education level.
